# The effect of inpatient rehabilitation programmes on quality of life in patients with cystic fibrosis: A multi-center study

**DOI:** 10.1186/1477-7525-4-8

**Published:** 2006-02-03

**Authors:** Tim G Schmitz, Lutz Goldbeck

**Affiliations:** 1 Department of Pediatrics, Schwabing, Technical University Munich, Köner Platz 1, 80804 München, Germany; 2 University Clinic of Child and Adolescent Psychiatry/Psychotherapy Ulm, Steinhövelstr. 5, 89075 Ulm, Germany

## Abstract

**Background:**

Disease-specific, multimodal inpatient rehabilitation programmes are designed to improve the physical, emotional, and social functioning of patients with cystic fibrosis (CF).

This study investigates the effects of a 4-week inpatient rehabilitation program on health-related quality of life (QL).

**Methods:**

84 German patients with CF (16–62 years of age, FEV1 mean 52.9% of the predicted), participating in an inpatient rehabilitation programme, completed the Questions on Life Satisfaction-Modules (FLZ^M^) at the beginning and one month after the end of intervention. The FLZ^M ^allows the multi-dimensional evaluation of subjectively perceived satisfaction with general and health-specific life domains. In addition, medical parameters (FEV1, Body Mass Index) and socio-demographic data were registered.

**Results:**

Compared to the baseline scores, after rehabilitation, participants were significantly more satisfied with general, health-related, and CF-related aspects of their lives. Specifically, improvement was noted in the following items: breathing difficulties/cough, sleep, integration of therapy into daily routine, leisure activities, general health perception, physical condition and being free from anxiety.

**Conclusion:**

Comprehensive inpatient rehabilitation programmes are a promising strategy to improve the quality of life of adolescent and adult CF-patients.

## Background

A severe and progressive disease such as cystic fibrosis (CF) is characterised by limited life expectancy and physical impairment. Furthermore, it imposes considerable emotional stress on the individual and requires intensive and time-consuming daily therapy, which may have adverse effects on quality of life (QL). Disease-specific inpatient rehabilitation programmes in Germany consider the multi-dimensionality of CF. The aim of these comprehensive interventions is not only to improve the patients' physical state of health and to optimize medical therapy, but also to provide emotional support, improve adherence to therapy, and facilitate social integration. In addition, efforts are made to maintain the patients' ability to work. Several specialized inpatient rehabilitation centres for patients with CF provide multi-modal disease-specific intervention programmes, including IV therapy if necessary, patient education, sports and training, nutritional counseling, physiotherapy and psychological support. Participants can meet other CF patients and get some relief from daily tasks and problems at home.

To date, there are few systematic evaluations of rehabilitation programmes for patients with CF. Results of a literature review via MEDLINE and PSYNDEX (a German language database), revealed only one study that investigated the effects of inpatient rehabilitation on CF patients' quality of life [1]. As no follow-up measurement was completed in that study, it is not clear whether the improvement of quality of life at the end of the rehabilitation programme was maintained in the home environment after treatment. So far, only a few studies have examined the effect of non-medical interventions in CF such as physiotherapy or exercise [2-5].

In a previous, study we demonstrated a high level of patient satisfaction with inpatient rehabilitation, however, no data on the programme's effect on QL were collected [6]. Adolescent and adult participants in this programme positively evaluated physiotherapy, communication with other CF patients, and the relief from the stress of their everyday problems. Currently, most interventions are evaluated in terms of their effects on pulmonary functioning, which does not provide information about other areas of functioning which are captured in QL measures. A central purpose of this study was to integrate a QL measure into the evaluation of this rehabilitation programme. Quality of life has been defined as a multidimensional construct, including physical, emotional and social functioning [7]. Correlations between objective health parameters, such as pulmonary function and QL are typically moderate and thus, integration of QL measures broadens the scope of the evaluation [8-10]. Because non-medical factors, such as coping with the disease, which are known to be correlated with QL [8], are addressed thoroughly in rehabilitation programmes, we expected to find positive effects of inpatient rehabilitation on the patients' QL.

The aim of this study was to investigate the effects of an inpatient rehabilitation programmes on CF patients quality of life after returning home.

## Methods

### Intervention

The duration of an individual inpatient rehabilitation, as defined by the German CF-Rehabilitation Consensus Group, is typically four to six weeks. Rehabilitation programmes in these centres include strict daily exercise training and physiotherapy (30 to 60 minutes), psychosocial support two to three times per week, nutritional counselling twice a week, patient education regarding physiotherapy, medical therapy and especially intravenous antibiotic therapy that is scheduled or needed for an exacerbation are compatible with international guidelines.

### Study design and sample

The study included a consecutive sample of adolescents and adults with CF (age > 15 years) participating in an inpatient rehabilitation programme at one of the seven German study sites. Approval was acquired from the local ethics committee at the study centre and informed consent from participants was collected by the physicians at the study sites. Data management was organised in collaboration between the study sites and the study centre. The assessment was done within the first three days of the intervention and 4–6 weeks after the end of the rehabilitation programme. At the first assessment, patients were instructed to evaluate their quality of life retrospectively for the four weeks prior to beginning the rehabilitation programme. The questionnaires were collected at the rehabilitation centre, checked for completeness and sent to the study centre. At discharge, the follow-up questionnaire was handed out to the patients with instructions on when to complete the second questionnaire at home. It was returned by participants via a stamped envelope, addressed to the study centre. Recent changes in health, reports on time spent doing therapy at home and socio-demographic data were recorded by the participants at both measurement points.

During enrolment, all 130 adolescent and adult rehabilitation patients with CF were asked to participate in the study. One hundred and six patients (81.5%) participated in the first assessment and 86 patients completed the follow-up questionnaire, returning it anonymously to the study centre (response rate: 81.1%). Two patients were excluded because of serious co-morbid diseases: a recently diagnosed carcinoma and severe depression. A comparison of the study group with those who did not participate or return the follow-up questionnaire revealed no significant differences in gender distribution (61 percent female) and body mass index (BMI; mean of 20.2). However, study participants were older (mean 29.0 vs. 26.6. years, *p *< 0.05) and had worse pulmonary functioning (forced expired volume in the first second (FEV_1_) 53 vs. 60.7 percent predicted, *p *< 0.01) than those who refused to participate at the study. A large percentage of patients in the programme (80.8%) were colonized with *pseudomonas aeruginosa*, with no cases of *burkholderia cepacia *detected. Finally, 39% of patients in our study were treated with intravenous antibiotic therapy during their stay at the rehabilitation centre.

### Measures

The Questions on Life Satisfaction FLZ^M ^[8,11,12] is a multi-dimensional instrument measuring general life satisfaction, satisfaction with health, and satisfaction with CF-specific aspects of life. The questionnaire consists of two 8-item modules and one 9-item module representing a broad range of domains. The respondent is asked to rate each item twice, first for the degree of subjective importance and secondly for his/her present degree of satisfaction in that domain. Life domains include *friends*, *leisure activities*, *occupation*, *living conditions*, *family life *or *partnership*. Satisfaction with health includes *physical condition*, *ability to relax*, *energy level *or *being free from anxiety*, and the CF-specific module assesses *breathing difficulties/cough*, *abdominal pain/digestive trouble*, *eating*, *sleep *or *integration of therapy into daily routine*.

The scales range from 1 (not important) through 2 (slightly important), 3 (moderately important), 4 (very important) to 5 (extremely important) for the importance ratings and in the same format from 1 (dissatisfied) through 2 (slightly dissatisfied), 3 (slightly satisfied), 4 (moderately satisfied) to 5 (very satisfied) for the satisfaction ratings. The two ratings are computed using a weighted satisfaction score: (*importance - 1*) × (*2 × satisfaction - 5*), yielding weighted satisfaction scores ranging from -12 to +20. With this transformation of raw scores to weighted satisfaction scores, negative scores indicate dissatisfaction and positive scores indicate satisfaction. Total scores for each of the three dimensions (general life satisfaction, satisfaction with health, CF-specific satisfaction) are calculated by summing the weighted satisfaction scores in the eight or nine domains for each dimension. Good reliability and validity of the FLZ^M ^have been previously reported [9,11].

In accordance with the targets of the inpatient rehabilitation programme, we examined the weighted satisfaction scores in the following domains: *leisure activities*, *general health*, *occupation *from the *General Life Satisfaction*-module; *physical condition*, *ability to relax*, *energy level*, *being free from anxiety *and *being free from discomfort and pain *from the *Satisfaction with Health*-module; *breathing difficulties/cough*, *abdominal pain/digestive trouble*, *eating*, *sleep*, *integration of therapy into daily routine*, *and consistency with daily therapy *for the CF-specific module. Additionally the three total scores for each module were used. In addition to the QL ratings, FEV_1_% predicted and BMI were recorded upon entering the rehabilitation programme and at the last visit to the outpatient CF-centre after discharge.

### Statistical procedures

Means and standard deviations were calculated for the weighted satisfaction scores in each of the relevant QL-domains mentioned above and for the three satisfaction summary scores. Paired *t*-tests were calculated for baseline- and follow-up scores. Bonferroni corrections of significance levels were performed for adjustment to multiple tests. We used the statistical programme SYSTAT 10.0 ^®^.

## Results

The average interval between the first and second was 55 days (range: 40 – 100 days) and missing data across assessments was low, approximately 2%.

After rehabilitation no patient showed a new colonisation with *pseudomonas aeruginosa*, *burkholderia cepacia *or *multiple resistant staphylococcus aureus *(MRSA). During the interval between the end of rehabilitation and the second assessment 12% of the patients reported intercurrent infection exacerbation. Socio-demographic data such as partnership, housing conditions or employment did not change between both assessments.

A domain with relatively high satisfaction at the beginning of the rehabilitation programme was *eating *(mean 8.0; SD 8.2). Patients were most dissatisfied in the domains of: *general health *(mean 1.4; *SD *7.4), *physical condition *(mean 1.7; *SD *7.0), *breathing difficulties/cough *(mean 2.2; *SD *8.3) and *occupation *(mean 2.3; *SD *6.8). Means, standard deviations and the results of paired *t*-tests for QL parameters, FEV_1_% and BMI are presented in Table 1.

**Table 1:** Comparison between quality of life scores and medical parameters, forced expiratory volume at one second (FEV1) and body mass index (BMI), at the beginning and one month after the end of rehabilitation programme by paired t-test Bonferroni adjusted (CI: .95 confidence interval of mean)

**Variable**	**T1 **mean (CI)/SD	**T2 **mean (CI)/SD	t	df	p
general life satisfaction	**39.6 **(31.8–48.5)/37.5	**45.8 **(38.0–53.8)/35.6	2.29	76	0.025
health-related life satisfaction	**55.2 **(46.1–64.6)/42.3	**63.1 **(54.5–72.7)/41.2	2.71	79	0.008
CF-related life satisfaction	**52.8 **(43.5–62.1)/42.4	**63.1 **(53.5–72.7)/43.6	3.21	81	0.002
FEV1% of the predicted	**52.9 **(48.5–58.0)/21.1	**55.5 **(50.8–60.1)/20.3	2.47	73	0.016
BMI	**20.2 **(19.6–20.8)/2.7	**20.5 **(19.9–21.0)/2.7	3.81	81	< 0.001

The effect of gender on improvement in QL was explored by separate paired *t*-tests for male and female patients. Men improve about twice as much as women (*General Life Satisfaction*: 9.3 vs. 4.2; *Satisfaction with Health*: 12.5 vs. 5.1; *CF-related life satisfaction*: 13.4 vs. 8.3). The effect of disease severity (mild: FEV1 > 70%; moderate: FEV1 40 – 70%; severe: FEV1 < 40%) on the improvement in QL was examined by repeated measures analyses of variance (ANOVAs). Only in *General Life Satisfaction *there was an effect of disease severity indicating that patients with moderate and severe manifestation of their disease improved significantly, whereas patients with mild pulmonary restriction did not improve.

**Figure 1:**
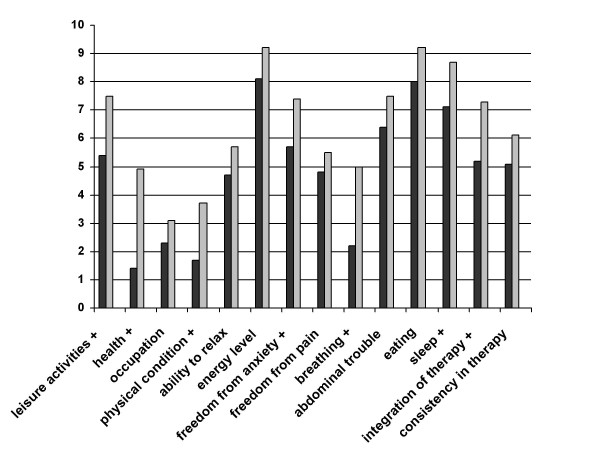
**Differential effects of the rehabilitation programme on quality of life: comparison of weighted satisfaction **(mean scores in the study group, scales ranging from -12 to +20) at the beginning (black) and one month after intervention (grey) in fifteen domains of the Questions on Life Satisfaction (FLZ^M^) (*N *= 84; + indicate significant improvement: p ≤ .05).

The intra-individual variations between the two assessments ranged from -51 to 73 (mean 6.9, *SD *23.9, *CI *of mean 1.4 – 12.4) for the *General Life Satisfaction *scale, from -55 to 88 (mean 8.8, *SD *26.1, CI 3.0 – 14.6) for the *Satisfaction with Health *scale and from -52 to 142 (mean 10.1, *SD *29.1, CI 3.6 – 16.5) for the *CF-related life satisfaction *scale. 49.3% of the patients experienced a relevant improvement, defined as scale improvement difference score higher as 4 points in the general life satisfaction scale, 51.2% in the satisfaction with health scale and 50.6% in the CF-related life satisfaction scale. The part of patients did not experienced a deterioration, defined as scale deterioration higher as -4, was 70,7% in the *General Life Satisfaction *scale, 68.7% in the *Satisfaction with Health *scale and 66.7% in the *CF-related life satisfaction *scale. Results of the paired *t*-tests revealed that the summary scores including general life satisfaction, health-related life satisfaction and CF-related life satisfaction of patients all improved significantly after rehabilitation. Detailed analyses showed that after rehabilitation seven of the 14 weighted satisfaction scores improved significantly: *breathing difficulties/cough *(5.0 vs. 2.2; *t *= 4.1; *df *= 82; *p *< .001), *sleep *(8.7 vs. 7.1; *t *= 2.0; *df *= 82; *p *= .05), *integration of therapy into daily routine *(7.3 vs. 5.2; *t *= 2.7; *df *= 81; *p *= .008), *leisure activities *(7.5 vs. 5.4; *t *= 3.5; *df *= 83; *p *= .001), *general health *(4.9 vs. 1.4; *t *= 4.8; *df *= 83; p < .001), *physical condition *(3.7 vs. 1.7; *t *= 3.1; *df *= 83; *p *= .02) and *being free from anxiety *(7.4 vs. 5.7; *t *= 2.1; *df *= 82; *p *= .04). All other weighted satisfaction scores also improved but the differences compared with the baseline scores were not statistically significant (Figure 1).

## Discussion

The results of this study indicate that the rehabilitation programme significantly improved both the objective health outcomes and the subjective quality of life outcomes of CF patients. Only minor effects on lung function and BMI occurred, however an improvement of 2.5% of the predicted in FEV_1 _or an increase in weight of some pounds can be an important step in a progressive disease such as cystic fibrosis.

Several domains of general and disease-specific quality of life were significantly improved in the study group, with strongest effects in satisfaction with *general health*, *breathing*, *physical condition*, *leisure activities*, and *integration of therapy into daily routines*. Because the intervention consisted of several components, it is not possible to identify any single element in the rehabilitation programme that was associated with these positive effects. Several components of the programme may have contributed, including intensification of medical therapy and physiotherapy, general physical recovery because of the reduction of daily stress, the presence of social and psychological support, improvements in patient adherence and education, and certain positive climatic influences at the study sites in the mountains or at the sea.

The observed improvement on the scale *therapy integration *indicates a decrease in conflicts between daily activities and time-consuming physiotherapy at home, whereas the satisfaction with *consistency in therapy *remained unchanged. It may be more difficult to change a patient's basic attitudes to his/her health situation and to the importance of adherence to therapy in a rehabilitation programme. No significant benefits were demonstrated for the abdominal system-complex, represented by the items *abdominal pain*/*digestive trouble *and *eating*. The stability in these domains may be due to a ceiling effect, as evidenced by the relatively high and stable satisfaction with this domain. A significant but modest improvement was found for BMI as objective indicator of weight-height relation, but this was not correlated with an improvement in satisfaction with eating.

Subgroup analyses demonstrated a significant gender effect on improvement of all three dimensions of life satisfaction. The higher benefit for men compared to women can be explained by the fact that the proportion of full-time working patients is twice as high among men. Especially working persons profit by a time-out and more opportunity for relaxation and physiotherapy. There may also be a general superiority of male patients in utilizing effective coping strategies, as indicated by a previous cross-sectional study [14] demonstrating that male patients with CF reported more life satisfaction than female patients. Compared to the clear gender effect, there was only a minor effect of disease severity, indicated by pulmonary function, on improvement of *General Life Satisfaction*. Only those patients with moderate or severe impairment of their pulmonary function responded positively to the rehabilitation program with regard to general aspects of their life as family, vocation and partnership. This may be due to a subjectively more relevant impairment in daily functioning prior to rehabilitation, and a subjectively perceived reconstitution of these daily functions as a consequence of rehabilitation. Further studies should investigate these correlations in detail.

Our findings replicate the positive effects shown previously by Staab et al. [1], however, this study demonstrated an improvement of QL even one month after the end of the rehabilitation programme. Thus, the positive effects on quality of life persisted after the end of rehabilitation. Our study may have underestimated the effects of the rehabilitation program on QL, considering the effect that patients might have already been influenced by positive expectations and anticipation of a stress-free time-out at the first assessment. Thus they may have reported a better QL at that time compared with the time before they arrived at the rehabilitation centre. Furthermore, the results of the second assessment might be influenced negatively by a rebound-effect, when the patients were once again confronted with their daily problems at home [13].

Future studies should consider how these effects might be enhanced or maintained in the home environment, where patients are once again faced with daily stressors and less psychological support. Differences between responders and non-responders to the intervention should be examined more detailed.

This study was limited in several respects and thus, interpretation of the results, while positive, should be viewed with caution. First, the small size of the study sample may have prevented the detection of further intervention effects due to limited statistical power. Secondly, a selection bias may have led to a more positive evaluation of the intervention by those who completed the post assessment, since it is possible that less satisfied patients may have refused to send back the follow-up questionnaire. Although anonymity of the data was assured, a tendency to answer in a socially desirable manner may have influenced our results. Negative feedback was provided by some patients, which may counter the notion that patients tended to answer in the socially desirable direction. Thirdly, for ethical and practical reasons we were not able to use a comparison group and to assign patients randomly to the intervention or a control condition. In Germany, the decision to participate in a rehabilitation programme is made by insurance companies and not by the patient him or herself. On the other hand, the naturalistic design can be considered a strength, because "real" patients were represented. Finally, the follow-up interval was quite short and the long-term effects of the intervention have not been investigated in this study.

## Conclusion

In summary, despite several methodical limitations, the results of this study indicate some evidence for the effectiveness of comprehensive inpatient rehabilitation programmes on the subjective health of adolescents and adults with CF. In the future, randomised controlled studies, component analyses and long-term follow-up studies should be conducted to obtain more evidence on the effects of inpatient rehabilitation programmes for CF.

## Authors' contributions

T.S. managed the study and coordinated the participated centres. Both authors participated in the design of the study and performed the statistical analysis, both drafted, read and approved the final manuscript.
